# Bioluminescent human breast cancer cell lines that permit rapid and sensitive *in vivo *detection of mammary tumors and multiple metastases in immune deficient mice

**DOI:** 10.1186/bcr1026

**Published:** 2005-04-08

**Authors:** Darlene E Jenkins, Yvette S Hornig, Yoko Oei, Joan Dusich, Tony Purchio

**Affiliations:** 1 Xenogen Corporation, Alameda, California, USA; 2 Currently employed by Chiron Corporation, Emeryville, California, USA

## Abstract

**Introduction:**

Our goal was to generate xenograft mouse models of human breast cancer based on luciferase-expressing MDA-MB-231 tumor cells that would provide rapid mammary tumor growth; produce metastasis to clinically relevant tissues such as lymph nodes, lung, and bone; and permit sensitive *in vivo *detection of both primary and secondary tumor sites by bioluminescent imaging.

**Method:**

Two clonal cell sublines of human MDA-MB-231 cells that stably expressed firefly luciferase were isolated following transfection of the parental cells with luciferase cDNA. Each subline was passaged once or twice *in vivo *to enhance primary tumor growth and to increase metastasis. The resulting luciferase-expressing D3H1 and D3H2LN cells were analyzed for long-term bioluminescent stability, primary tumor growth, and distal metastasis to lymph nodes, lungs, bone and soft tissues by bioluminescent imaging. Cells were injected into the mammary fat pad of nude and nude-beige mice or were delivered systemically via intracardiac injection. Metastasis was also evaluated by *ex vivo *imaging and histologic analysis postmortem.

**Results:**

The D3H1 and D3H2LN cell lines exhibited long-term stable luciferase expression for up to 4–6 months of accumulative tumor growth time *in vivo*. Bioluminescent imaging quantified primary mammary fat pad tumor development and detected early spontaneous lymph node metastasis *in vivo*. Increased frequency of spontaneous lymph node metastasis was observed with D3H2LN tumors as compared with D3H1 tumors. With postmortem *ex vivo *imaging, we detected additional lung micrometastasis in mice with D3H2LN mammary tumors. Subsequent histologic evaluation of tissue sections from lymph nodes and lung lobes confirmed spontaneous tumor metastasis at these sites. Following intracardiac injection of the MDA-MB-231-luc tumor cells, early metastasis to skeletal tissues, lymph nodes, brain and various visceral organs was detected. Weekly *in vivo *imaging data permitted longitudinal analysis of metastasis at multiple sites simultaneously. *Ex vivo *imaging data from sampled tissues verified both skeletal and multiple soft tissue tumor metastasis.

**Conclusion:**

This study characterized two new bioluminescent MDA-MB-231-luc human breast carcinoma cell lines with enhanced tumor growth and widespread metastasis in mice. Their application to current xenograft models of breast cancer offers rapid and highly sensitive detection options for preclinical assessment of anticancer therapies *in vivo*.

## Introduction

Development of breast cancer mouse models that provide consistent primary mammary tumors and metastasis to clinically relevant tissues such as lymph nodes, lungs, and bone remain a challenge in the preclinical evaluation of therapies for human breast cancer. Current xenograft models of breast carcinoma involve murine or human breast cancer cell lines implanted into the mammary fat pad of mice or injected systemically by intravenous or intracardiac routes. Tumor cells injected into the mammary tissue yield reproducible tumors, but can require weeks to several months for primary tumor development and produce varied spontaneous metastasis depending on the cell line and mouse strain used in the study [1].

One common human breast cancer cell line used in xenograft animals models is MDA-MB-231. These cells originated from a human metastatic ductal breast carcinoma sample [2], are estrogen independent, and exhibit preferential growth in the mammary fat pad of immune compromised mice [3]. MDA-MB-231 cells develop primary tumors that produce spontaneous metastasis to lymph nodes and micrometastases to the lungs [4]. Detection of metastasis has relied primarily upon histological or PCR analysis of selected tissues at experimental end-points. Spontaneous metastasis to bone or soft organs from primary mammary tumors has not been reported.

Reproducible bone metastasis in breast cancer xenograft models has been achieved with intracardiac injection of MDA-MB-231 cells [5,6]. Passaging tumor cells harvested from the bone lesions several times *in vivo *has created MDA-MB-231 sublines with exclusive propensity for bone metastasis [7-10]. The bone metastases are typically identified in animals by radiographic or histological procedures. Recently, researchers have begun to apply luciferase-based imaging methods to detect widespread metastasis in mouse breast cancer models [10-13]. In studies using luciferase-expressing MDA-MB-231 tumor cell sublines specifically selected for skeletal metastasis, *in vivo *imaging was able to monitor experimental bone metastasis in mice to a level comparable to that of X-ray analysis [10,13]. Our goal was to develop a bioluminescent human breast cancer cell line that would offer a similar level of detection for both primary and metastatic tumors and would more fully mimic clinical breast cancer by metastasizing to multiple tissues, including lymph nodes, lungs, bone, and visceral organs.

This report describes bioluminescent xenograft mouse models based on more widely metastatic derivatives of MDA-MB-231 cells. These two luciferase-expressing cell lines, D3H1 and D3H2LN, were isolated for stable firefly luciferase expression *in vitro *and were passaged in mice to enhance their tumorigenic and metastatic properties. We evaluated the effect of long-term *in vivo *growth on the stability of cellular bioluminescence. *In vivo *and *ex vivo *imaging was used to monitor and compare the primary tumor growth rates and metastatic potential from mammary tumors and after intracardiac injection of the two MDA-MB-231 sublines over time.

## Materials and methods

### Tumor cell line

Human breast cancer cell line MDA-MB-231 was obtained from the American Type Culture Collection (Rockville, MA, USA). Cells were cultured in Minimum Essential Medium with Earl's Balanced Salts Solution MEM/EBSS medium supplemented with 10% fetal bovine serum, 1% nonessential amino acids, 1% L-glutamine, and 1% sodium pyruvate (all from Hyclone, Logan, UT, USA) at 37°C in a humidified atmosphere containing 5% carbon dioxide.

### Co-transfection and selection of cell line

MDA-MB-231 cells were co-transfected with plasmids expressing the firefly luciferase gene (*luc*; pGL3-Red, Chris Contag, Stanford University, Stanford, CA, USA) and zeocin resistance gene (pSV40/Zeo; Invitrogen, Carlsbad, CA, USA) using lipofectamine (Invitrogen). Transfected cells were then selected for antibiotic resistance (zeocin; Invitrogen) and surviving colonies were screened for bioluminescence in complete media supplemented with 150 μg/ml D-luciferin (Biosynth International, Inc, Naperville, IL, USA) by *in vitro *imaging using the IVIS™ camera system (Xenogen, Alameda, CA, USA; see below). Bioluminescent, antibiotic resistant, single cell clones were amplified in culture and characterized for stable luminescence *in vitro *and tumorigenic potential *in vivo*. One clonal cell line, MDA-MB-231-luc-D3, was initially selected. This D3 clone was passaged through mice as a primary tumor in the mammary fat pad to produce the D3H1 cell line. A spontaneous lymph node metastasis from a D3H1 mammary fat pad tumor was also harvested and designated D3H2LN. These three bioluminescent derivatives of MDA-MB-231 cells were used for further *in vitro *or *in vivo *studies.

### Bioluminescent imaging

Bioluminescent imaging was performed with a highly sensitive, cooled CCD camera mounted in a light-tight specimen box (IVIS™; Xenogen), using protocols similar to those described previously [14-17]. Imaging and quantification of signals were controlled by the acquisition and analysis software Living Image^® ^(Xenogen). For *in vitro *imaging, bioluminescent cells were serially diluted from 4000 to 8 cells in complete media into black, clear bottomed, 96-well plates (Costar, Acton, MA, USA). D-luciferin (Biosynth International, Inc.) at 150 μg/ml in media was added to each well 5–10 min before imaging. Imaging time was 1 min/plate. For *in vivo *imaging, animals were given the substrate D-luciferin by intraperitoneal injection at 150 mg/kg in DPBS Dulbecco's Phosphate Buffered Saline (Invitrogen, Carlsbad, CA, USA), and anesthetized (1–3% isoflurane). Mice were then placed onto the warmed stage inside the light-tight camera box with continuous exposure to 1–2% isoflurane. Imaging times ranged from 1 s to 3 min, depending on the tumor model and time point. Generally, two to three mice were imaged at a time. The low levels of light emitted from the bioluminescent tumors or cells were detected by the IVIS™ camera system, integrated, digitized, and displayed. Regions of interest from displayed images were identified around the tumor sites and were quantified as total photon counts or photons/s using Living Image^® ^software (Xenogen). Background bioluminescence *in vivo *was in the region of 1 × 10^4 ^photon counts or 1–2 × 10^5 ^photons/s. For *ex vivo *imaging, 150 mg/kg D-luciferin was injected into the mice just before necropsy. Tissues of interest were excised, placed into 24-well tissue culture plates with 300 μg/ml D-luciferin in DPBS, and imaged for 1–2 min. Tissues were subsequently fixed in 10% formalin (Sigma, St. Louis, MO, USA) and prepared for standard histopathology evaluation.

### Mouse strain and animal care

Strict animal care procedures set forth by the Institutional Animal Care and Use Committee based on guidelines from the US National Institutes of Health Guide for the Care and Use of Laboratory Animals were followed for all experiments [18]. Mice used in these studies were either female athymic nude-nu mice aged 8–10 weeks (Harlan, Indianapolis, IN, USA) or female nude-beige (NIH-bg-nu-xidBR) mice aged 8–10 weeks (Charles River Laboratories, Wilmington, MA, USA).

### Mammary fat pad spontaneous metastasis model

Female nude mice or female nude-beige mice aged 8–10 weeks were anesthetized by exposure to 1–3% isoflurane and injected with 50 μl of 2 × 10^6 ^MDA-MB-231 cells suspended in 50% DPBS/50% matrigel into the abdominal mammary fat pad. At 10–15 min after luciferin injection, mice were placed in the IVIS™ Imaging System and imaged from the ventral view. Tumor growth was monitored weekly by bioluminescent imaging and external caliper measurements (tumor size = [length × width × height] × 0.52) for 5–9 weeks. In some experiments, the lower portion of each animal was shielded before reimaging in order to minimize the bioluminescence from the primary tumor so that the signals from the metastatic regions could be observed *in vivo*. The front limbs were secured with tape to better expose the axillary/brachial lymph node areas. The imaging time ranged from 1 s to 1 min, depending on the size of the primary tumors, but was consistently 3 min for detection of metastases.

### Intracardiac experimental metastasis model

Female nude mice (age 8–10 weeks) were anesthetized by intramuscular injection of 120 mg/kg ketamine hydrochloride with 6 mg/kg xylazine on the day of injections, and by exposure to 1–3% isoflurane on subsequent imaging days. On day 0, anesthetized animals were injected with 1 × 10^5 ^MDA-MB-231 cells suspended in 100 μl sterile DPBS into the left ventricle of the heart by nonsurgical means [19]. Anesthetized mice were placed in the IVIS™ Imaging System and imaged from both dorsal and ventral views approximately 10–15 min after intraperitoneal injection of D-luciferin. A successful intracardiac injection was indicated on day 0 by images showing systemic bioluminescence distributed throughout the animal. Only mice with evidence of a satisfactory injection continued in the experiment. Assessment of subsequent metastasis was monitored *in vivo *once a week by imaging for up to 5 weeks.

### Histopathology

To confirm the presence of neoplastic cells, selected tissues were excised from the mice at necropsy and were preserved in 10% formalin solution (Sigma) immediately after *ex vivo *imaging. Tissues were prepared for histopathology (paraffin preparation, sectioning, and hematoxylin and eosin staining) and analyzed by subsequent microscopic evaluation by IDEXX Veterinary Services (West Sacramento, CA, USA).

### Statistical analyses

The mean bioluminescence (photons/s), tumor volume, and corresponding standard errors of the mean were determined for each experiment. Regression plots were used to describe the relationship between bioluminescence and cell number and tumor volume; R^2 ^values are reported to assess the quality of the regression model.

## Results

### Stable luciferase expression of MDA-MB-231-luc derivative cell lines

Parental MDA-MB-231 cells were transfected with a plasmid encoding firefly luciferase, and several stable clones were selected *in vitro*. A resulting bioluminescent cell line with reasonable bioluminescence *in vitro *(D3) was isolated and injected into the mammary fat pad of nude mice. Following 12 weeks of growth *in vivo*, tumor cells were harvested from a primary tumor and re-propagated *in vitro *to produce the MDA-MB-231 subclone line D3H1. These cells were injected once more into the mammary fat pad of mice to yield a second cell line, D3H2LN, harvested from a lymph node metastasis of a D3H1 mammary tumor.

**Figure 1:**
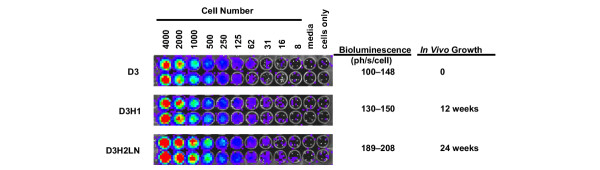
*In vitro *bioluminescence of MDA-MB-231 sublines D3, D3H1 and D3H2LN. Cells from each subline were serially diluted in duplicate wells from 4000 to 8 cells/well. Luciferin substrate was added to each well and the plate was imaged to obtain photons/s per cell. Wells with media (no cells) or cells alone (no luciferin) were included as controls. The range of bioluminescence for each subline represents data collected from two to three experiments.

To determine whether prolonged growth *in vivo *affected bioluminescence of the D3H1 or D3H2LN cells over time relative to the original D3 luciferase-expressing line, serial dilutions from cell cultures of each were imaged and compared *in vitro *(Fig. 1). The mean photon emissions ranged from approximately 100 to 208 photons/s per cell, and we found no decrease in bioluminescence as the MDA-MB-231 luciferase-expressing subclones were passaged through animals. The D3H2LN cells were in fact slightly brighter *in vitro *than the D3H1 or original D3 cells. The data indicate that the level of luciferase expression remained relatively stable for up to 12 weeks of tumor growth for the D3H1 cells, and after an additional 12 weeks of *in vivo *growth for the D3H2LN cells.

### Enhanced tumorigenicity of D3H1 and D3H2LN cell lines in the mammary fat pad

**Figure 2:**
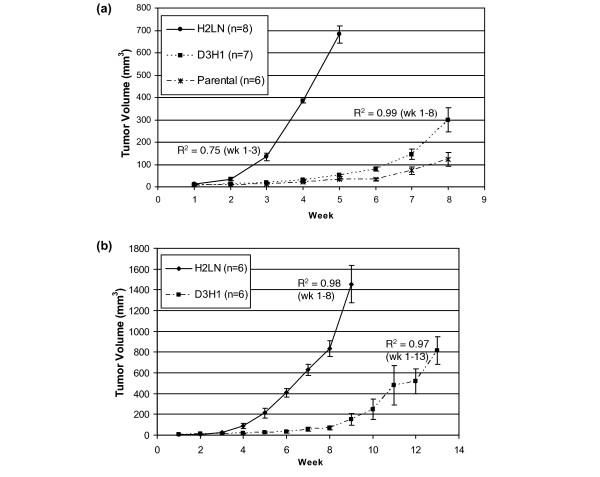
Mammary tumor growth is enhanced in D3H1 and D3H2LN bioluminescent sublines of MDA-MB-231 cells. Cells were injected into the mammary fat pad of **(a) **nude or **(b) **nude-beige mice, and mean tumor volumes were determined by caliper measurements over time. The tumors from the bioluminescent sublines D3H1 and D3H2LN were also monitored using *in vivo *imaging, and the correlation between mean photons/s and mean tumor volume are indicated as R^2 ^values.

The mammary tumor growth rates of the two luciferase-expressing derivatives were compared with the nonglowing parental MDA-MB-231 cells (Fig. 2a). Mean external caliper measurements of mammary tumors indicated that the D3H1 cells grew at a slightly faster rate than did the original parental cells. The D3H2LN cells exhibited a dramatic increase in tumor growth relative to both the D3H1 and the parental cells *in vivo*. By 5 weeks of growth in nude mice, the D3H2LN tumors had reached a volume more than two to seven times larger than the tumors of the other cell lines at 8 weeks after injection (Fig. 2a). Similar results were found with nude-beige mice (Fig. 2b), although tumor growth in general was slower for both D3H1 and D3H2LN cells in these experiments as compared with the rates in nude mice.

The growth of D3H1 and D3H2LN mammary tumors in nude or nude-beige mice was also monitored *in vivo *by bioluminescent imaging. The mean photons emitted from the tumors over time in each cohort were compared with the corresponding tumor volumes of the same animals. Bioluminescent data correlated with tumor volume, with overall R^2 ^values of 0.95–0.98 (Fig. 2a, b). The only exception occurred at the final time points for the D3H2LN cells in nude mice, when the extremely fast growing tumors at weeks 4 and 5 continued to increase in volume but the bioluminescence leveled off or decreased. The developing necrotic cores in these tumors, confirmed by postmortem histological analysis (histology data not shown), presumably contributed to the diminished photon emission at the later time points.

### Spontaneous metastasis from primary mammary fat pad tumors

**Figure 3:**
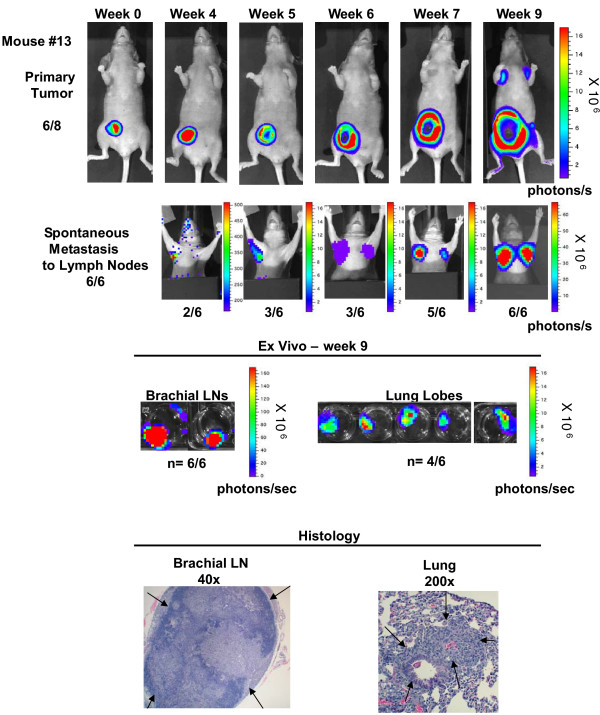
D3H2LN mammary tumors grow rapidly and produce bilateral lymph node metastasis. Bioluminescent D3H2LN cells were injected into nude-beige mice, and tumor growth and metastasis were monitored over time *in vivo*. Data for a representative mouse are shown. Tumor take rate was high (6/8 mice) and lymph node metastasis to both the right and left thoracic areas was detected by week 9 in all mice with mammary tumors (upper panels). *Ex vivo *images of excised brachial lymph nodes and lung lobes are shown (middle panels). Histological analysis of the same tissues confirmed perfuse metastasis to the lymph nodes and isolated micrometastasis to the lung lobes (lower panels).

Mice with D3H1 and D3H2LN mammary tumors were evaluated *in vivo *for spontaneous metastasis to the lungs or thoracic lymph nodes by shielding the bright primary tumors growing in the abdominal fat pad and reimaging the thorax of each animal. Representative images over time from a nude-beige mouse with D3H2LN tumors are presented in Fig. 3. A summary of the data from nude and nude-beige mice with D3H1 or D3H2LN tumors is presented in Table 1.

Similar to the D3H2LN tumor volume data shown in Fig. 2b, D3H2LN mammary tumor bioluminescence started to increase about 4 weeks after cell injection in nude-beige mice (Fig. 3, upper panels). Metastatic signals also appeared at that time in the right axillary region in 33% (2/6) of the mice, indicating spontaneous thoracic metastasis on the same side of the animal as the developing distal primary tumor. Subsequent thoracic images demonstrated an incremental increase in metastatic growth over time, and by week 6 all six mice had developed right axillary signals. Also at this time point, half of the mice exhibited additional metastatic signals in the left axilla, and by the end of the experiment all animals had developed bilateral thoracic metastases (Fig. 3).

The location and size of the *in vivo *bioluminescent signals suggested lymph node metastasis, and so the brachial and axillary lymph nodes of all mice were excised at necropsy, imaged *ex vivo*, and preserved for histological evaluation. Lung lobes were also included in the analysis as an alternative site for metastasis. Figure 3 (middle panel) shows the *ex vivo *images of the lymph nodes and lung lobes from a representative mouse. Brachial lymph nodes in all mice and lung lobes in 67% (4/6) of the cohort were bioluminescent *ex vivo*. The brighter *ex vivo *signal of the right brachial lymph nodes compared with the left lymph nodes correlated with the relatively higher intensity observed *in vivo *in the right thoracic area at the final time points. In the lung lobes, the *ex vivo *signal was at least 10-fold lower than the brachial lymph nodes, suggesting a much lower tumor burden in lungs than in lymph nodes. Subsequent histological evaluation confirmed extensive and pervasive tumor growth in the brachial lymph nodes and limited, sporadic micrometastasis in the lung lobes (Fig. 3, lower panels). Selected axillary lymph nodes, which were negative for bioluminescence *ex vivo*, were also found to be negative by histological examination (data not shown).

### Tumor growth and metastasis of D3H2 tumors versus D3H2LN tumors

The mammary tumor take rate was lower and metastatic tumor growth was slower in studies with D3H1 cells than in those with D3H2LN cells. Table 1 shows the results for both nude and nude-beige mice. Metastatic spread to lymph nodes was also more limited in mice with D3H1 primary tumors, and spontaneous metastasis to both right and left brachial lymph nodes was rare or nonexistent. In contrast, all mice with D3H2LN mammary tumors developed right lymph node metastasis, and detection of bilateral spread was found in 100% of the nude beige mice. The detection of micrometastasis to lung lobes was observed in 38–67% of the mice with D3H2LN mammary tumors, whereas lung micrometastasis was nearly absent in mice that had D3H1 mammary tumors. Overall, nude-beige mice had higher frequencies of lung or lymph node metastasis than did nude mice, regardless of the tumor cell type used in the study.

### Detection of metastases following intracardiac injection of MDA-MB-231-luc cells

**Figure 4:**
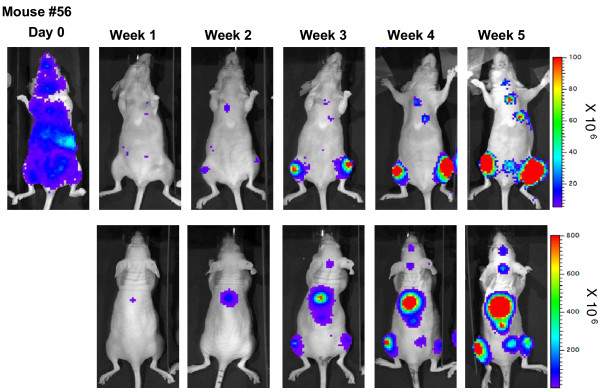
Multiple metastases detected *in vivo *following intracardiac injection of D3H2LN cells. Ventral (upper panels) and dorsal (lower panels) images taken over time from a representative nude mouse injected with D3H2LN cells are shown. Pseudocolor scale bars were consistent for all images of ventral or dorsal views in order to show relative changes at metastatic sites over time.

We used a nonsurgical intracardiac injection of D3H1 or D3H2LN cells to compare experimental metastasis after systemic circulation of cells. Mice were imaged immediately after injection of cells and once a week thereafter. Ventral and dorsal images of a representative nude mouse injected with D3H2LN cells are shown in Fig. 4. Animals with successful intracardiac injection (8/12) on day zero showed low level, widespread bioluminescence minutes after the injection. Within 2 weeks, localized bioluminescent signals indicating metastasis began to appear at a few sites in each animal. By 3–4 weeks after injection, all eight mice exhibited clear indications of tumor growth at multiple sites in the head, thorax, abdomen, legs, or spine. None of these tumors was superficially obvious or palpable.

**Figure 5:**
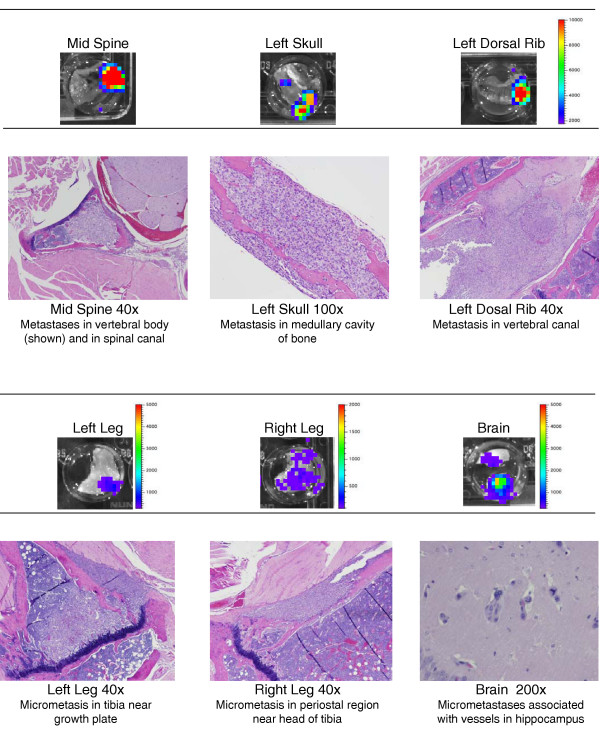
*Ex vivo *and histological data confirm metastases from intracardiac injection of D3H2LN cells. Representative data from selected tissues from two mice that were evaluated by both *ex vivo *imaging and subsequent histological analysis are shown.

*Ex vivo *imaging was conducted with 17–20 different tissues excised from each mouse after the final *in vivo *imaging session at week 5. These tissues included untrimmed specimens of bones (jaw, skull, ribs, scapulae, clavicles, femur/tibia, vertebrae) along with isolated lungs, lymph nodes, salivary glands, and soft tissues such as brain, liver, pancreas, uterus, kidney and adrenals. In general, *ex vivo *images corroborated widespread metastasis in all mice injected with D3H2LN cells (Table 2). Representative *ex vivo *images of skeletal and brain metastasis are shown in Fig. 5, along with views of their corresponding histology sections. In all mice (8/8) metastasis to some type of bone-associated tissue was detected by *ex vivo *imaging, with frequencies greater than 50% for spine, skull, scapula, leg, or clavicle. A majority of animals injected with D3H2LN cells exhibited bioluminescent evidence of metastasis to the rib cage (8/8), brain (7/8), lungs (6/8), and thoracic lymph nodes (5/8). Less frequent metastasis (2–4/8) was observed *ex vivo *in the liver, pancreas, kidney/adrenals, uterus, heart, or salivary glands.

The panel of tissues examined by *ex vivo *imaging was also evaluated by histological analysis in two of the eight mice that presented with widespread metastasis from D3H2LN cells. Tumor lesions were sporadic and small in many of the tissues and some had to be re-sectioned and evaluated two or three times in order to locate what often was described as a few micrometastases per section. Nevertheless, metastasis was identified by microscopic examination in the majority of the tissues from at least one of the two mice sampled (Table 2, Fig. 5). In nearly all the lungs and rib sections, micrometastases were detected on the pleural surface, and not within the lung parenchyma or bone proper. It was generally not possible to detect micrometastasis to the larger soft organs by histology in the tissues from the two mice examined, although *ex vivo *imaging detected low levels of bioluminescence (data not shown).

Mice injected with D3H1 cells exhibited widespread metastasis *in vivo *after intracardiac injection but with lower frequency at each site compared with animals injected with the D3H2LN cells (Table 2). The first *in vivo *indication of metastasis in any of the D3H1 cohort occurred at week 5, which was 3 weeks later than in animals injected with the same number of D3H2LN cells (Fig. 5), and it took 10 weeks for metastasis to develop in a majority of animals injected with D3H1 cells versus 5 weeks for mice injected with D3H2LN cells. Although metastasis was confirmed by histology in most of the tissue samples from animals with D3H1 cells, isolated micrometastasis was frequently found associated with attendant muscle or fat rather than within the organs themselves (data not shown).

## Discussion

Bioluminescent imaging has been used to detect primary cancer growth and metastasis in a growing number of animal models [14-20]. In xenograft tumor models, human cell lines expressing luciferase have permitted studies that yield real-time, noninvasive monitoring of tumor sites in the same cohort of animals over time. This study is the first application of *in vivo *bioluminescent imaging to monitor breast cancer tumor growth in animals and to detect spontaneous metastasis of tumor cells from the mammary fat pad to lymph nodes and lungs. The luciferase-expressing subclones of MDA-MB-231 characterized in this report also produced multiple metastases at high frequencies to clinically relevant tissues such as bone and brain following intracardiac injection of cells. With the ability to produce lymph node and lung metastasis from primary tumors and widespread metastasis after intracardiac injection, the D3H1 and D3H2LN subclones of MDA-MB-231 cells fully mimic the range of breast cancer development in humans.

Previous studies have noted lymph node and lung metastasis from parental MDA-MB-231 mammary tumors in mice but have largely relied on postmortem histology or PCR to document the presence of limited metastasis, especially to the lungs, from MDA-MB-231 primary tumors [4]. Our imaging experiments yielded similar findings but they did not require time consuming tissue processing and analysis. The low bioluminescence of the micrometastasis to the lung from mammary tumors was detected immediately in excised tissues by *ex vivo *analysis. The more pervasive lymph node metastasis was detected in both live animals over time and by *ex vivo *examination. Subsequent microscopic evaluation of the metastatic tissues initially identified as positive or negative by bioluminescent imaging was used to confirm tumor spread and allowed precise localization and sizing of lesions at the cellular level.

MDA-MB-231 luciferase-expressing cells were injected in the mammary fat pad of mice and passaged one or two times *in vivo *to generate a more robust xenograft model of human breast cancer with enhanced primary tumor growth and subsequent metastasis. In evaluating the photon emission of these cell lines, we found no decrease in bioluminescence between the D3H1 cells, D3H2LN cells, and the original unpassaged D3 subclone from the parental MDA-MB-231 cells, despite 12 weeks of tumor growth for D3H1 cells and 24 weeks of accumulated *in vivo *growth time for the D3H2LN line. Additionally, the D3H1 and D3H2LN cell lines maintained consistent levels of bioluminescence *in vitro *for up to 2 months or approximately eight to 10 passages in culture (data not shown). These data demonstrate remarkable long-term stability of luciferase expression after multiple rounds of *in vivo *growth and continuous periods of *in vitro *culturing.

The intracardiac injection model is used to generate widespread arterial dissemination of tumor cells by bypassing the lungs in order to seed cells to various organs. This method has been employed with luciferase-positive MDA-MB-231 derivative cell lines that were selected for enhanced *in vivo *metastasis to bone [10,13]. The metastatic spread of these cells after intracardiac injection was primarily to bone sites, with limited visceral metastasis to the adrenals or pancreas. Our D3H1 and D3H2LN sublines expand the utility of such models by producing consistent metastatic lesions to brain and various visceral organs as well as skeletal sites following intracardiac injection. In previous reports researchers compared bioluminescent imaging with radiometric measurements of bone metastasis, and found *in vivo *imaging to be the faster and more sensitive detection method [10]. Similarly, our intracardiac experiment compared imaging with histological examination and showed that, in many cases, tissues that had been found to be positive by *in vivo *or *ex vivo *imaging required multiple rounds of sectioning and histology to identify the micrometastasis.

In both nude and nude-beige mice, the D3H2LN tumors produced a faster rate of tumor growth in the mammary fat pad as well as enhanced spontaneous metastasis to the lymph nodes and lungs compared with the D3H1 cells (Fig. 2 and Table 1). The D3H2LN cells also exhibited higher frequencies of metastasis to bone, brain, lungs, and soft tissues after intracardiac injection (Table 2). Taken together, these findings suggest a more invasive phenotype in the D3H2LN cells and demonstrate an enhanced utility in animal metastasis studies. We did find that the rapid growth of D3H2LN cells in some mammary fat pad experiments created necrotic tumors that showed a decrease in bioluminescent signal *in vivo *at later time points, despite an incremental increase in tumor volume (Fig. 2a). A similar disparity between volume measurements and bioluminescence with larger tumors was reported in a study using subcutaneous tumors of human prostate cells expressing luciferase [11]. Here, the authors noted that tumor volume became static after treatment of animals, while a drop in bioluminescence of the same tumors indicated a tumoricidal effect on cells that was subsequently confirmed by histology.

## Conclusion

The bioluminescent stability of the D3H1 and D3H2LN cell lines, coupled with their enhanced tumorigenicity and widespread metastatic potential, provides a sensitive *in vivo *model system for preclinical assessment of breast cancer growth, dissemination, and response to anticancer therapies.

## Abbreviations

DPBS = Dulbecco's Phosphate Buffered Saline; PCR = polymerase chain reaction.

## Competing interests

All authors are current or former employees of Xenogen Corporation. Xenogen manufactures the IVIS Imaging System™ and provides commercially available bioluminescent tumor cell lines as part of their Bioware™.

## Authors' contributions

DEJ conceived the study, directed the experiments, and wrote the manuscript. YSH and YO carried out the *in vivo *experiments, organized the data, and contributed to drafts of the manuscript. JD carried out *in vitro *experiments and participated in drafting the final manuscript. TP supervised the project and participated in drafting the manuscript.

## Figures and Tables

**Table 1:** Mammary fat pad model tumor take and metastasis

Cell line	Experimental parameters				Detected metastases	
	
	Mouse strain	*n*	Take rate	End-point (weeks)	LN *in vivo*	Lung *ex vivo*
D3H1	Nude	9, 10	40–78%	8–13	75–86% R, 0% L	0–16%
	Nude-beige	6	66%	13	67% R, 16% L	0%
D3H2LN	Nude	9, 8	89–100%	5	100% R, 0% L	38%
	Nude-beige	8	75%	9	100% R, 100% L	67%

L, left; LN, lymph node; R, right.

**Table 2:** *Ex vivo *detection of metastasis after intracardiac injection (nude mice)

	H2LN (*n *= 8)	D3H1 (*n *= 5)
Experimental end-point	Week 5	Week 10
Skeletal metastasis	8/8	4/5
Spine	88%^a^	60%^a^
Skull	75%^a^	40%^a^
Scapula	75%	40%^a^
Femur/tibia	63%^a^	0%^a, b^
Clavicle	50%	0%
Upper/lower jaw	13–23% ND	0–20%
Ribs and associated tissues	8/8^a^	4/5^a^
Brain	7/8^a^	2/5^a^
Lungs	6/8^a^	2/5^a^
Lymph nodes (axillary/brachial)	5/8^a^	2/5
Other soft tissues (heart, liver, pancreas, kidney, adrenals, uterus, salivary gland)	4/8	2/5

^a^Metastasis confirmed by histology in at least one animal sample. ^b^Three out of five mice exhibited *in vivo *leg signal, and metastasis was confirmed by histology. ND, tissue not analyzed by histology.

## References

[B1] Price JE (1996). Metastasis from human breast cancer cell lines. Breast Cancer Res Treat.

[B2] Cailleau R, Young R, Olive M, Reeves WJ (1974). Breast tumor cell lines from pleural effusions. J Natl Cancer Inst.

[B3] Price JE, Polyzos A, Zhang RD, Daniels LM (1990). Tumorigenicity and metastasis of human breast carcinoma cell lines in nude mice. Cancer Res.

[B4] Rose DP, Connolly JM, Liu XH (1994). Effects of linoleic acid on the growth and metastasis of two human breast cancer cell lines in nude mice and the invasive capacity of these cell lines in vitro. Cancer Res.

[B5] Yoneda T, Sasaki A, Mundy GR (1994). Osteolytic bone metastasis in breast cancer. Breast Cancer Res Treat.

[B6] Yoneda T (1997). Arterial microvascularization and breast cancer colonization in bone. Histol Histopathol.

[B7] Yoneda T, Williams PJ, Hiraga T, Niewolna M, Nishimura R (2001). A bone-seeking clone exhibits different biological properties from the MDA-MB-231 parental human breast cancer cells and a brain-seeking clone in vivo and in vitro. J Bone Miner Res.

[B8] Michigami T, Hiraga T, Williams PJ, Niewolna M, Nishimura R, Mundy GR, Yoneda T (2002). The effect of the bisphosphonate ibandronate on breast cancer metastasis to visceral organs. Breast Cancer Res Treat.

[B9] Kang Y, Siegel PM, Shu W, Drobnjak M, Kakonen SM, Cordon-Cardo C, Guise TA, Massague J (2003). A multigenic program mediating breast cancer metastasis to bone. Cancer Cell.

[B10] Wetterwald A, van der Pluijm G, Que I, Sijmons B, Buijs J, Karperien M, Lowik CW, Gautschi E, Thalmann GN, Cecchini MG (2002). Optical imaging of cancer metastasis to bone marrow: a mouse model of minimal residual disease. Am J Pathol.

[B11] Mendel DB, Laird AD, Xin X, Louie SG, Christensen JG, Li G, Schreck RE, Abrams TJ, Ngai TJ, Lee LB (2003). In vivo antitumor activity of SU11248, a novel tyrosine kinase inhibitor targeting vascular endothelial growth factor and platelet-derived growth factor receptors: determination of a pharmacokinetic/pharmacodynamic relationship. Clin Cancer Res.

[B12] Murray LJ, Abrams TJ, Long KR, Ngai TJ, Olson LM, Hong W, Keast PK, Brassard JA, O'Farrell AM, Cherrington JM (2003). SU11248 inhibits tumor growth and CSF-1R-dependent osteolysis in an experimental breast cancer bone metastasis model. Clin Exp Metastasis.

[B13] Minn AJ, Kang Y, Serganova I, Gupta GP, Giri DD, Doubrovin M, Ponomarev V, Gerald WL, Blasberg R, Massague J (2005). Distinct organ-specific metastatic potential of individual breast cancer cells and primary tumors. J Clin Invest.

[B14] Jenkins DE, Oei Y, Hornig YS, Yu SF, Dusich J, Purchio T, Contag PR (2003). Bioluminescent imaging (BLI) to improve and refine traditional murine models of tumor growth and metastasis. Clin Exp Metastasis.

[B15] Jenkins DE, Yu SF, Hornig YS, Purchio T, Contag PR (2003). In vivo monitoring of tumor relapse and metastasis using bioluminescent PC-3M-luc-C6 cells in murine models of human prostate cancer. Clin Exp Metastasis.

[B16] Scatena CD, Hepner MA, Oei YA, Dusich JM, Yu SF, Purchio T, Contag PR, Jenkins DE (2004). Imaging of bioluminescent LNCaP-luc-M6 tumors: a new animal model for the study of metastatic human prostate cancer. Prostate.

[B17] Rehemtulla A, Stegman LD, Cardozo SJ, Gupta S, Hall DE, Contag CH, Ross BD (2000). Rapid and quantitative assessment of cancer treatment response using in vivo bioluminescence imaging. Neoplasia.

[B18] Arguello F, Baggs RB, Frantz CN (1988). A murine model of experimental metastasis to bone and bone marrow. Cancer Res.

[B19] Contag CH, Jenkins D, Contag PR, Negrin RS (2000). Use of reporter genes for optical measurements of neoplastic disease in vivo. Neoplasia.

[B20] (1996). Institute of Laboratory Animal Resources (ILAR) Guide for the Care and Use of Laboratory Animals:.

